# Pathway and Molecular Mechanisms for Malachite Green Biodegradation in *Exiguobacterium* sp. MG2

**DOI:** 10.1371/journal.pone.0051808

**Published:** 2012-12-14

**Authors:** Ji’ai Wang, Feng Gao, Zhongzhong Liu, Min Qiao, Xuemei Niu, Ke-Qin Zhang, Xiaowei Huang

**Affiliations:** Laboratory for Conservation and Utilization of Bio-Resources, and Key Laboratory for Microbial Resources of the Ministry of Education, Yunnan University, Kunming, Yunnan, China; University of Kansas, United States of America

## Abstract

Malachite green (MG), N-methylated diaminotriphenylmethane, is one of the most common dyes in textile industry and has also been used as an effective antifungal agent. However, due to its negative impact on the environment and carcinogenic effects to mammalian cells, there is a significant interest in developing microbial agents to degrade this type of recalcitrant molecules. Here, an *Exiguobacterium* sp. MG2 was isolated from a river in Yunnan Province of China as one of the best malachite green degraders. This strain had a high decolorization capability even at the concentration of 2500 mg/l and maintained its stable activity within the pH range from 5.0 to 9.0. High-pressure liquid chromatography, liquid chromatography-mass spectrometry and gas chromatography–mass spectrometry were employed to detect the catabolic pathway of MG. Six intermediate products were identified and a potential biodegradation pathway was proposed. This pathway involves a series of reactions of N-demethylation, reduction, benzene ring-removal, and oxidation, which eventually converted N-methylated diaminotriphenylmethane into N, N-dimethylaniline that is the key precursor to MG. Furthermore, our molecular biology experiments suggested that both triphenylmethane reductase gene *tmr* and cytochrome P450 participated in MG degradation, consistent with their roles in the proposed pathway. Collectively, our investigation is the first report on a biodegradation pathway of triphenylmethane dye MG in bacteria.

## Introduction

Biodegradation refers to chemical decomposition of organic substances by living organisms or other biological means. One major purpose of microbial research in this field is to identify environmental microorganisms with significant activities to degrade recalcitrant organic substances and further investigate their related pathways. Depending on the knowledge of biodegradative pathways as well as the underlying molecular mechanisms, genetic methods can also be used to construct efficient microbial strains with versatile and high degradation abilities. Similarly, multi-functional biocatalyst may be created using the immobilized enzyme technology, which could be an alternative strategy to manage comprehensive ecological problems. For example, the aerobic biodegradation of polycyclic aromatic hydrocarbons (PAHs) by microorganisms is a key process for eliminating these toxic compounds from polluted soils and sediments [1]. Meanwhile, the discovery of bacterial degradation to nitroaromatic compounds, which are among the largest and most important groups of industrial chemicals in use today though many of them are acutely toxic, mutagenic, and carcinogenic, is also demonstrated a potential treatment for environmental contamination [2].

Malachite green (MG) is one type of triphenylmethane dyes and is extensively used in both the textile industry and the fish farming industry due to its relatively low cost, ready availability, and high efficacy against fish microbial pathogens. Despite its high value, MG is highly toxic to mammalian cells, and has been banned by the US Food and Drug Administration [3]. Meantime, the insufficient treatment of wastes from industries can also lead to environmental problems [4]. Thus several different methods have been explored to decontaminate wastewater and protect the environment [5]. Developing microbial biodegradation agents is an important approach for solving these problems due to their effective cost, friendliness to the environment, and small quantities of sludge [6], [7].

**Figure 1:**
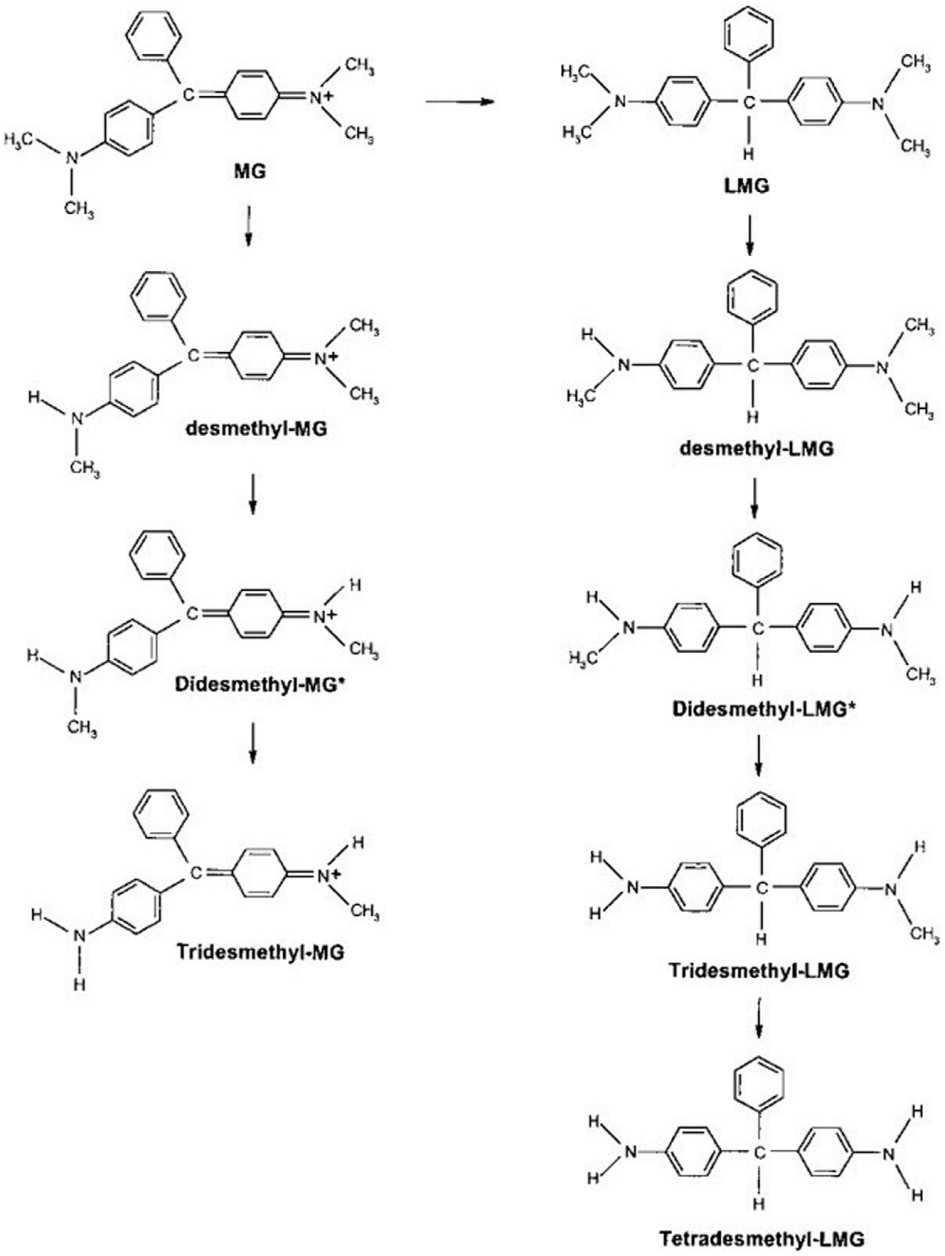
The proposed biodegradation pathway for MG in the Fungus *Cunninghamella elegans* (from reference 3).

The recalcitrant MG and its cytotoxicity present difficulties for biodegradation. However, several microbial strains have been reported capable of decolorizing MG. These microorganisms include the white rot fungus *Phanerochaete chrysosporium*, three birds’ nest fungi such as *Cyathus bulleri*, *Cyathus stercoreus*, and *Cyathus striatus*, as well as some bacterial strains such as *Achromobacter xylosoxidans* MG1, *Bacillus cereus* DC11, *Aeromonas hydrophila* DN322, *Pseudomonas otitidis* W/L3, *Mycobacteria*, *Citrobacter strain* KCTC 18061P, *Mycobacteria* and a strain from *Kurthia* sp. [7]–[13]. As for the metabolic pathway(s) and genes involved MG degradation, an incomplete biodegradation pathway of MG with triphenylmethane as the end-product has been partially described in the fungus *Cunninghamella elegans*. In this pathway, the reactions included reduction and N-demethylation, and produced tridesmethyl MG or tridesmethyl leucomalachite green (LMG) (figure 1) [3]. In bacterial decomposition of MG, the substrate is only known to transform into colorless LMG by an enzyme triphenylmethane reductase (TMR) [14]. Additionally, other extracellular enzymes, such as lignin peroxidase (LIP), laccase and manganese peroxidase (Mnp) from white rot fungi, have been reported to be involved in MG biodegradation, but the catalysis is a non-specific oxidizing reaction to crystal violet or other triphenylmethane dyes [6]. The role of cytochrome P450 in MG decolorization has also been confirmed in strains of *Mycobacterium chelonae*, *M. avium* and the fungus *C. elegans* [3], [9].

Despite progress so far in this area, our knowledge about biodegradation of MG or other triphenylmethane dyes is still in its infancy. Especially, no pathway for MG biodegradation has been completely elucidated in any microorganisms. Here, we investigated MG decolorizing activity in the bacterium *Exiguobacterium* sp MG2. The members of genus *Exiguobacterium* have never been reported to decolorize MG. The experimental data from high-pressure liquid chromatography (HPLC), liquid chromatography-mass spectrometry (LC-MS) and gas chromatography–mass spectrometry (GC-MS) led us to propose a potential catabolic pathway for the degradation of triphenylmethane dyes in bacteria. During biodegradation, a series of reactions, including N-demethylation, reduction, benzene ring-removal, and oxidation, drastically converted N-methylated diaminotriphenylmethane into N, N-dimethylaniline that is a key precursor to MG. Furthermore, evidences from molecular biology experiments also partially supported the proposed pathway.

## Materials and Methods

### 1. Ethics Statement

No specific permits were required for the described field studies. No specific permissions were required for these locations/activities. The location is not privately-owned or protected in any way.

### 2. Isolation and Identification of Bacterial Strains for Decolorizing Dye

The bacterial strains capable of decolorizing dye were screened from river water samples in a city of Mile, Yunnan Province, China. MG and crystal violet used in this study were of analytical grade and purchased from Kermel Co. The screening of optimal bacterial strains for dye decolorization was carried out on enrichment agar plates containing (g/l): yeast extract 5, NaCl 10, tryptone 10, agar 20, and MG 50 mg/l. The bacterial identification was mainly based on 16S rDNA sequences amplified by PCR using two universal primers as follows: P(f) 5′ AGAGTTTGATCCTGGCTCAG 3′; P(r) 5′ GGTTACCTTGTTACGACTT 3′ [15]. The reaction conditions were 35 cycles of 94°C 30 s, 56°C 30 s, and 72°C 90 s. Lastly, the 16S rDNA gene produced from PCR was sequenced and analyzed by comparing to those related sequences in GenBank.

### 3. Assay of MG Decolorization

Dye decolorization was monitored by the decrease of absorbance at 617 nm, the maximum wavelength of MG. The samples were prepared as described by Wang et al [13]. Briefly, bacterial cells were aerobically cultured in 250 ml ﬂasks containing 90 ml LB medium at 38°C with shaking at 190 rpm overnight. A moderate concentration 40 mg/l of MG was added into 1 ml cultured cells. After decolorization for 30 min or 2 h, bacterial cells were removed by centrifugation at 12,000 rpm for 2 min, and then the supernatant samples were used for absorbance measurement. The activity was determined by percentage decolorization. Decolorization rate (%) = 100 (A – B)/A, in which A represented initial absorbance and B represented observed absorbance after treatment [16]. All assays were performed in triplicates and the data shown here were the averages of three repeats.

### 4. Factors Influence the Efficacies of MG Decolorization

The assays to determine the effect of different factors that influenced the efficacies of decolorization were performed based on previous studies [12], [13]. After bacterial cells were aerobically cultured under the same situations until cell density reached OD_600_ 0.50–0.60, the cultures with 10^9^ cells/ml were added to various concentrations of MG and incubated for 3 h. After incubation, decolorization rates were calculated using the same method as described above. The tested factors included different concentrations of substrate and pH environments. In experiments to detect the effect of MG concentration on the rate of decolorization, sterile MG was added to a final concentration of 1000 mg/l, 1500 mg/l, 2000 mg/l and 2500 mg/l, respectively. To test the effect of pH on decolorization, the cultures were respectively adjusted to pH 5.0, 6.0, 7.0, 8.0, 9.0 by using HCl or NaOH.

### 5. Identification of the Intermediates in MG Biodegradation by HPLC, LC-MS and GC-MS

For HPLC, the cells of *Exiguobacterium* sp. MG2 were first induced into the resting stage. During the induction, bacterial cells were aerobically cultured in 250 ml ﬂasks containing 100 ml LB medium at 38°C with shaking at 190 rpm overnight, followed by centrifugation to remove the supernatant at 8,000 rpm and 4°C for 10 min. After washing in 50 mM PBS (pH 7.0) for three times, the preparation of testing cells was completed. Then, 6 ml resting cells or the negative control of 6 ml 50 mM PBS were added into the 100 ml MG solution with the concentration of 40 mg/l. When the decolorization rates in the test group reached about 90%, the cells was removed by centrifugation at 8,000 rpm for 20 min, and the supernatant was concentrated to 1 ml by rotary evaporator at 50°C and then dissolved by 1 ml methanol. Another negative control included the supernatant of resting cell without the substrate MG to cross reference the peaks of extracellular compounds. The operation conditions of the mobile phase were ammonium acetate (50 mM) from 70% to 5% in 50 min, and acetonitrile from 30% to 95% within 50 min, the detector wavelengths at 230 nm, 254 nm, 297 nm, 357 nm and 617 nm, the flow rate at 0.8 ml/min, and the LC column from ZORBAX SB-C18 (4.6×250 mm, 5 µm Agilent) at 35°C temperature. The injection volume was 10 µl.

The same samples were used in the LC-MS (G1969A LC/MSD TOF). The mobile phase was water and acetonitrile. The initial acetonitrile proportion was 6% ramped gradually to 35% in 15 min, then to 60% in 20 min, finally reached 95% in 15 min, and then held for 8 min. The ion source was ESI and the injection volume was 15 µl, with 190 nm to 780 nm spectrum range.

Before the GC-MS assay (Agilent 7890A/5975C, USA), the samples described above were further concentrated by evaporation and then dissolved into 2 ml methanol. The temperature program of the column was 100°C to 280°C at a rate of 6°C/min. The reaction intermediates were identified by comparing their spectra with those of the standards. The split ratio was 10∶1.

### 6. Inhibition of Dye Decolorization by Metyrapone

The involvement of cytochrome P450 in dye decolorization was determined by examining the effect of metyrapone, a known inhibitor of the enzyme. Cellular membrane and soluble fractions were prepared as described before [13], [17]. After the centrifugation of fermentation broth, the pellet was washed in 50 mM PBS (pH 7.0) containing 1.0 mM EDTA. Cells were disrupted by sonication in an ice bath (on 5 s, off 7 s, time 15 min, 48% amplitude) and MgCl_2_ was added to a final concentration of 2.0 mM. The crude extract fraction was obtained by centrifugation at 12,000 rpm for 1 min at 4°C, and the supernatant fraction was centrifuged again at 12,000 rpm for 30 min at 4°C to separate the cytoplasmic membrane (pellet) and soluble (supernatant) fractions. Lastly, the cellular membrane, soluble fractions, as well as the mixture of them were added to a metyrapone solution respectively to a final concentration of 10 mM. After incubation at 37°C for 10 min, the dye MG was added to a final concentration of 10 mg/l. The changes in decolorization rates were determined again. The negative control was the same volume of ddH_2_O instead of metyrapone.

### 7. Analysis of the *tmr* Gene

To identify if *tmr*, the gene responsible for decolorizing triphenylmethane dyes in *Citrobacter* sp. KCTC 18061P [14], also existed in the *Exiguobacterium* sp. MG2, a pair of PCR primers were designed based on the reference sequence GenBank AY756172. The primers were P1 (f) 5′ GATAGGAGGCATTCACCTTG 3′ and P1 (r) 5′ AGACTCTATGGATGCGCGC 3′ [14]. The reaction conditions were 35 cycles of 94°C 30 s, 56°C 30 s, and 72°C 50 s. After PCR completed, the amplicon was sequenced and further compared with those in the GenBank.

### 8. Statistical Analysis

All the data are expressed as mean values±SD. Comparisons among multiple groups were made with a one-way analysis of variance (ANOVA) followed by Dunnet t test. *P*≤0.05 was used to determine statistical significance of the observed differences between treatments.

The Genbank accession numbers for the 16S rRNA gene and *tmr* gene in *Exiguobacterium* sp. MG2 are JX436461 and JX436462, respectively.

## Results

### 1. Identification of MG Decolorizing Bacterium and its Decolorization Activities

Compared with other tested strains, an isolated bacterial strain yielded the best growth and decolorization activity on the enrichment medium containing 50 mg/l MG. When the sequence of its 16S rRNA gene was compared with those in GenBank, it showed a 99% similarity to the known sequences from *Exiguobacterium* sp. But since 16S rRNA genes do not discriminate among *Exiguobacterium* species, the isolate was designated *Exiguobacterium* sp. MG2. Our strain has been submitted to the China General Microbiological Culture Collection Center as CGMCC 4476.

**Figure 2:**
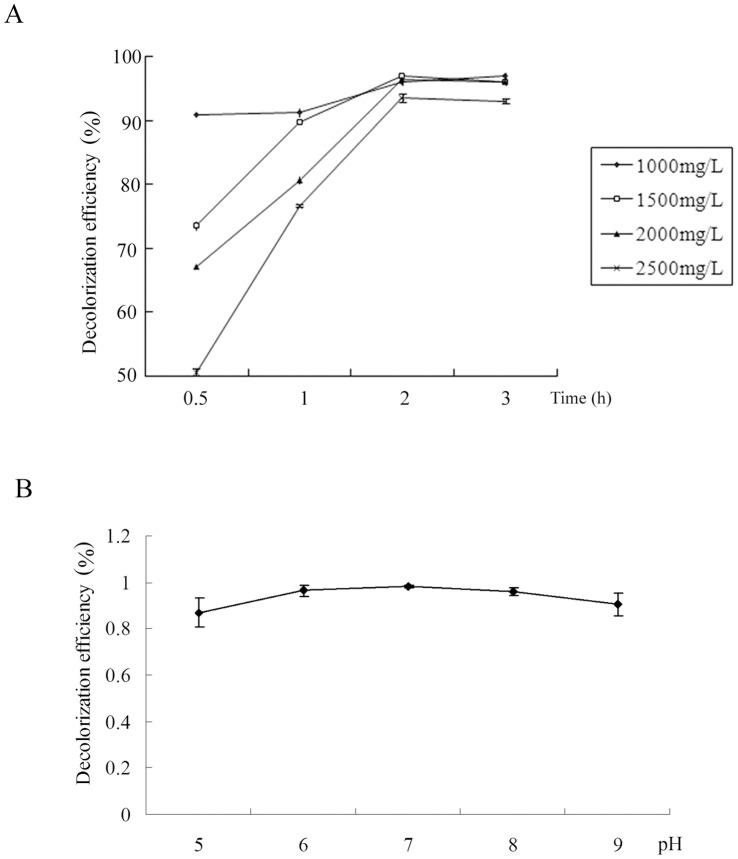
Effects of MG concentration and pH on decolorization efficiencies in strain *Exiguobacterium* sp. MG2. (A) Effects of MG concentration. The x values represented different concentrations of malachite green, and y values represented decolorization efficiencies. (B) Effects of pH. The x values were the tested pH environments, and y values were decolorization efficiencies.

Further quantitative analysis demonstrated that this strain could maintain high MG decolorizing activities, for MGs in concentrations ranging from 1000 mg/l to 2500 mg/l. When the dye concentration reached 2500 mg/l, this bacterium still showed 93.50±0.1% decolorizing efficiency within 2 h, the highest decolorizing and tolerance abilities to MG reported so far. It should be noted that the efficiencies of dye decolorization reduced tremendously with the increase of MG concentration at the initial stage. As time went on, however, this decrease gradually became unnoticeable (figure 2A). Furthermore, *Exiguobacterium* sp. MG2 could decolorize crystal violet, though the decolorization efficiency was much lower than that of malachite green decolorization (data not shown).

Our data also showed little effects on decolorization efficiencies within the assayed pH values. From pH 5.0 to 9.0, the strain’s MG decolorizing activity was about 90% of the maximum efficiency (figure 2B).

### 2. Detection to Intermediate Products and the Probable Pathway of MG Degradation

**Figure 3:**
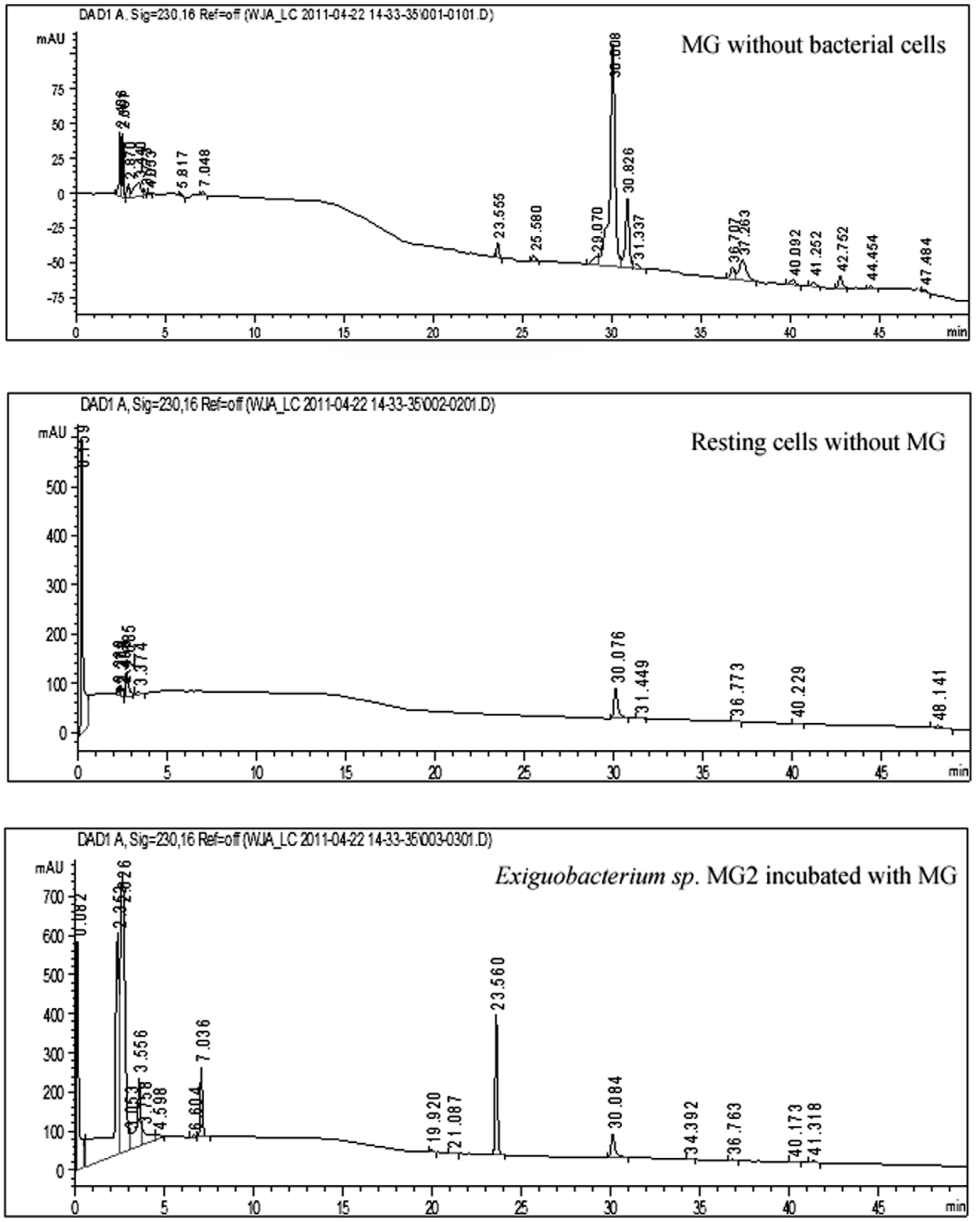
The results of HPLC analyses of catabolic intermediates. The first two maps were from the controls including MG without the resting cells and the resting cells without MG. The third one represented the result of *Exiguobacterium* sp. MG2 incubated with MG.

**Figure 4:**
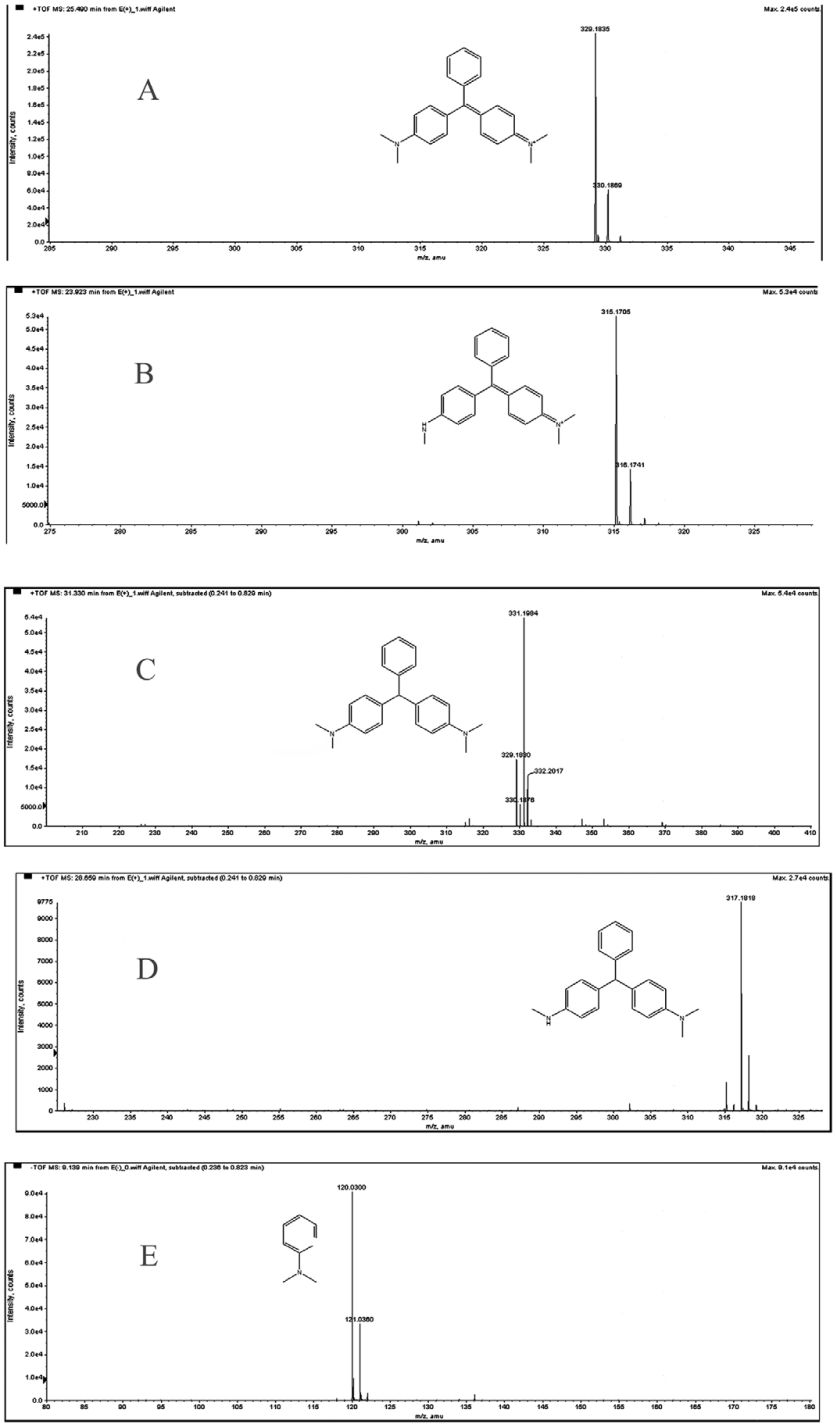
Selected ion chromatograms of LC-MS for metabolites derived from the biodegradation of MG in *Exiguobacterium* sp. MG2. (A) m/z 329, malachite green (retention time 25.543 min); (B) m/z 315, desmethyl malachite green (retention time 23.976 min); (C) m/z 331, leucomalachite green (retention time 31.330 min); (D) m/z 317, desmethyl leucomalachite green (retention time 28.659 min); (E) m/z 120, N,N-dimethylaniline (retention time 9.139 min). The small peaks below the main ones were the isotope effect.

**Figure 5:**
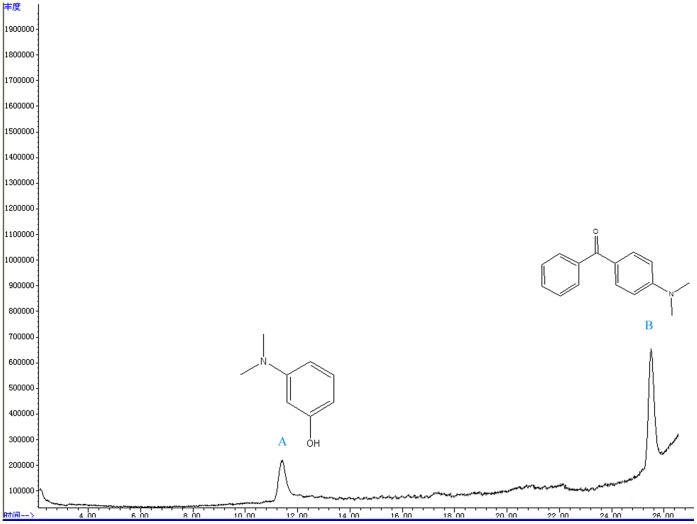
Selected chromatograms of GC-MS for metabolites derived from the biodegradation of MG in *Exiguobacterium* sp. MG2. The peak of A was 3-dimethylamino-phenol and the peak of B was (4-dimethylamino-phenyl)-phenyl-methanone, both of which were dissolved in methanol. As for the LMG, it was detected in the solvent of ethanol and thus was not shown here.

During incubation, the loss of MG color suggested that the dye was transformed to its leuco- form [3]. To confirm this hypothesis, the metabolites from resting cells of *Exiguobacterium* sp. MG2 incubated with MG were analyzed. HPLC, LC-MS and GC-MS were employed to detect the intermediate products. From the results of HPLC, we obtained several specific peaks of candidate intermediate products that were clearly distinguished compared with the controls of MG without bacterial cells or only resting cells without MG. These peaks were found at retention times 2.352, 2.626, 7.036, 19.920, 21.087 and 23.560 (figure 3). Due to the limitations of HPLC, we still lacked enough deterministic data to confirm the categories of those substances. Therefore, LC-MS was used and four possible intermediate products were identified according to the m/z based on a previous report [18]. These peaks corresponded to MG (m/z 329) and its mono-derivatives (m/z 315), LMG (m/z 331) and its mono-derivatives (m/z 317). The ion polarities of these compounds were all positive. While another substance with the m/z 120 of negative ion polarity, we consider it as N, N-dimethylaniline using the software Analyst QS and ChemDraw (figure 4). Considering the potential biases for detecting compounds among assays, the intermediates were further identified by GC-MS. From the GC-MS data, three metabolites were detected, which were LMG, (4-Dimethylamino-phenyl)-phenyl-methanone and 3-Dimethylamino-phenol respectively (figure 5).

Combining the results from HPLC, LC-MS and GC-MS, six products including LMG, desmethyl MG or LMG, (4-Dimethylamino-phenyl)-phenyl-methanone, 3-Dimethylamino-phenol and N, N-dimethylaniline were identified (table S1). The results suggest that biodegradation of MG involves a series of reactions of N-demethylation, reduction, benzene ring-removal, and oxidation.

### 3. Cloning of the Gene *tmr*

In a previous study of bacterial biodegradation of MG, the enzyme TMR was shown to be responsible for the conversion of MG to LMG [14]. Here, a PCR reaction was used to isolate the gene *tmr* from *Exiguobacterium* sp. MG2. After amplification with genomic DNA as template, a specific amplicon of 691 bp was obtained, as we had expected. Furthermore, the amplified gene of *tmr* showed 98% identity to that in *Citrobacter* sp. KCTC 18061P. This result suggests that triphenylmethane reductase TMR in *Exiguobacterium* sp. likely perform a similar function, contributing to the reduction reaction during decolorization of MG.

### 4. Inhibition of Metyrapone on Decolorization

Cytochrome P450 is a large and diverse group of enzymes, most of which functions to catalyze the oxidation of organic substances, drugs and other toxic chemicals. Because cytochrome P450 has been reported to be involved in MG degradation of *Mycobacteria* [9], [19], metyrapone, an inhibitor of cytochrome P450, was used to determine its impact on MG decolorization in our strain. Our results demonstrated that the decolorization efficiency from the membrane fraction was about 42.9±1.5% after adding metyrapone compared to 69.8±2.1% in the negative control that was added the same volume of ddH_2_O instead of metyrapone; while the decolorization efficiency from cytoplasm was about 3.7±0.7% while the negative control had the decolorization efficiency of 19.8±1.2%. This result suggested that the metyrapone had an obvious inhibitory effect on the decolorization in strain *Exiguobacterium* sp. MG2, and that the mono-oxygenase cytochrome P450 likely participated in the biodegradation of MG.

## Discussion

Many investigations on bacteria, including the strains such as *Pseudomonas otitidis* W/L3, *Achromobacter xylosoxidans* MG1, *Citrobacter* sp. KCTC 18061P, have been performed in the field of MG biodegradation. At present, their biodegradation pathways as well as the involved mechanisms still remained unclear [12]–[14]. Among all of the previously described bacterial strains, *A. xylosoxidans* MG1 had a very high efficiency in MG decolorization. It had been shown to reduce 88.50% of 2,000 mg/l MG within 2 h under an optimal condition [13]. However in our current study, *Exiguobacterium* sp. MG2, the first strain in this genus that has been reported to degrade MG, can remove 93.50% of the color within the same time at the concentration of 2,500 mg/l dye. Furthermore, this bacterial strain has a very stable decoloring activity within the broad pH range; while in *A. xylosoxidans* MG1, different pHs showed significantly different results. For these reason, *Exiguobacterium sp.* MG2 is the best MG degrader reported so far.

**Figure 6:**
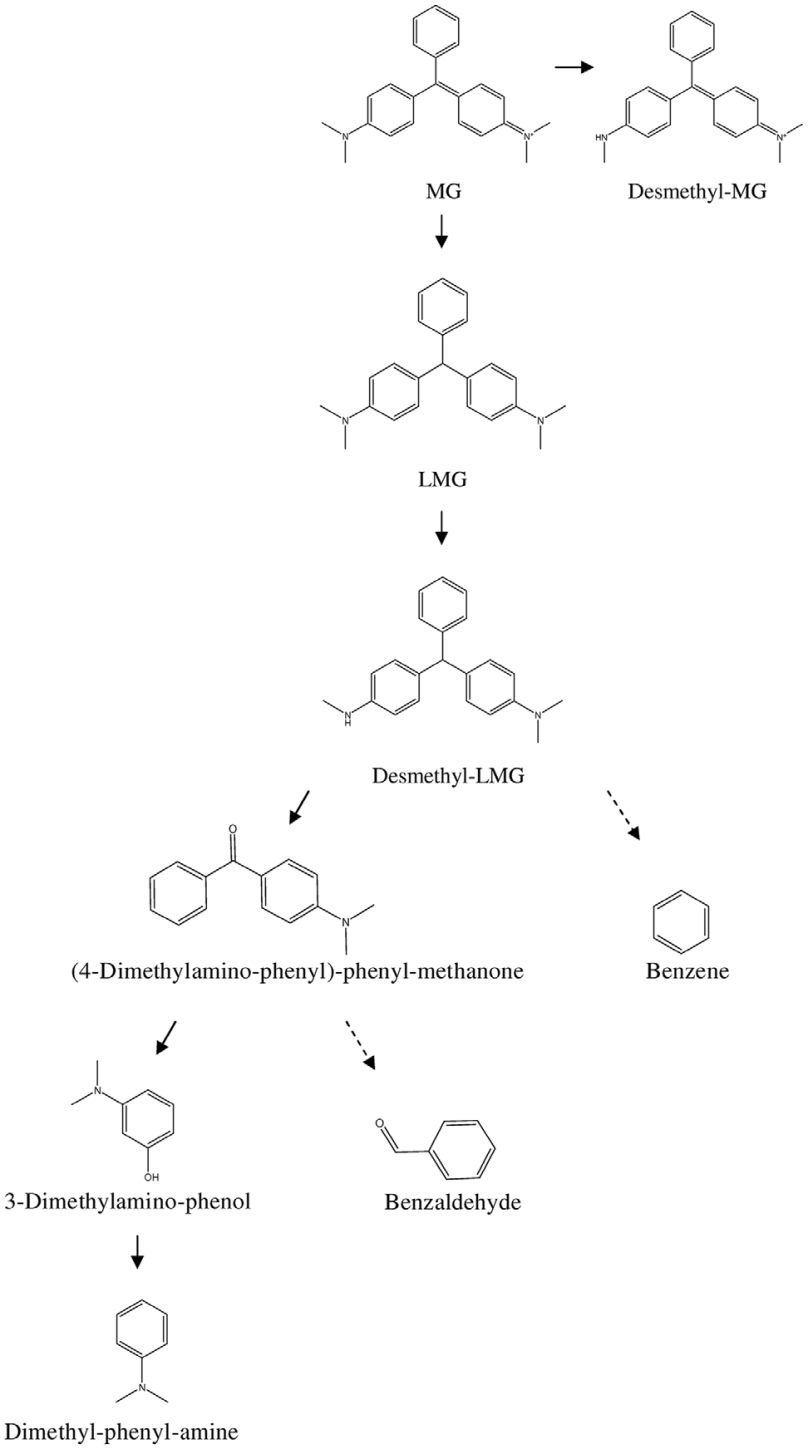
The proposed pathway of MG degradation in *Exiguobacterium* sp. MG2. The substances under dotted arrow were not detected in our LC-MS or GC-MS analyses, while the solid arrow represented the intermediate products detected in our experiments.

To the knowledge on biodegradation pathway of triphenylmethane dyes, crystal violet degradation by *B. subtilis* IF0 13719 and *Nocardia corallina* seemed similar to each other that included the breakage of triphenylmethane structure, and the presence of Michler’s Ketone and α-dimethylaminophenol as the major end-products [16], [20]. However, either tridesmethyl MG or LMG, the end-products in MG degradation by *C. elegan*, keeps the intact triphenylmethane structure [3], [21]. Therefore, we here tried to map a more comprehensive biodegradation pathway in strain *Exiguobacterium* sp. MG2. Based on the metabolites determined in our above experiments, we speculate that MG degradation in this strain at least involves the reactions of N-demethylation, reduction, benzene ring-removal, and oxidation. Lastly, we propose a MG degradation pathway for *Exiguobacterium sp.* MG2 (figure 6). In this pathway, the desmethyl-MG is derived form N-demethylation of MG, which also occurs in the transformation of desmethyl-LMG from LMG in a later step. The reductive reaction is involved in the formation of LMG, an leuco form of MG. Benzene ring-removal can be found in desmethyl-LMG’s cleavage to produce (4-dimethylamino-phenyl)-phenyl-methanone and benzene, which also involves the reaction of oxidation and the breaking of the C–C bond. Again, the breaking of the C–C bond results in the emergence of 3-dimethylamino-phenol and benzaldehyde. Finally, N, N-dimethylaniline formation requires the reaction of hydroxy-removal. Analysis to the contribution of each reaction in MG transformation suggests that the reductive reaction is a key step to change the dye into its colourless form. Additionally, the reaction of benzene ring-removal together with oxidation achieves the breakage of the triphenylmethane structure. And since the end-product N, N-dimethylaniline from the last step of hydroxy-removal is the industrial raw material of MG, it is theoretically reasonable to reuse it in MG production.

Both PCR amplification to the *tmr* gene and the inhibition of metyrapone on decolorization suggested that the enzyme TMR and cytochrome P450 are involved in MG dye decolorization in *Exiguobacterium* sp. MG2. In the postulated pathway, the first step transforming MG to LMG is through the enzyme of TMR, whose function is well reported to catalyze MG into its leuco form [14]. Cytochrome P450 most likely participated in the production of desmethyl-MG and desmethyl-LMG, as has been shown in the fungus *C. elegans* [3]. In addition, similar mechanisms might exist in the formation of 3-dimethylamino-phenol and benzaldehyde in MG biodegradation by *Exiguobacterium* sp. MG2 as those involved in the production of Michler’s Ketone and α-dimethylaminophenol during crystal violet degradation by *B. subtilis* IF0 13719 or *N. corallina*. However, the genes or enzymes responsible for oxidation, benzene ring-removal, or hydroxy-removal in *B. subtilis* IF0 13719 and *N. corallina* require further elucidation because no information about the corresponding genes or enzymes is known.

In conclusion, this report is the first to propose a pathway about MG biodegradation in bacteria. In addition, we have partially elucidated the probable molecular mechanisms. This study should help future understanding of the complete biodegradation mechanisms of MG or other triphenylmethane dyes with similar structures. Furthermore, the widely distributed bacterium *Exiguobacterium* sp. MG2 is nonpathogenic and has the highest efficiency known in the decoloration of MG. If more genes or enzymes involved in MG biodegradation were elucidated, this strain as well as its pathway could be a candidate host from which to construct a very useful ‘super bacterium’ for degrading recalcitrant chemicals.

## Supporting Information

Table S1 Summary of intermediate products during MG degradation by *Exiguobacterium* sp. MG2 as revealed by LC-MS and GC-MS.
(DOC)
