# Supplementary material for: Pathway and Molecular Mechanisms for Malachite Green Biodegradation in Exiguobacterium sp. MG2
Source: PLoS One. 2012 Dec 14;7(12):e51808. doi: 10.1371/journal.pone.0051808 (PMC3522578; doi:10.1371/journal.pone.0051808)
Supplement: Table S1 — Summary of intermediate products during MG degradation by Exiguobacterium sp. MG2 as revealed by LC-MS and GC-MS. (DOC) [file pone.0051808.s001.doc]

***Table S1*** *Summary of intermediate products during MG degradation by Exiguobacterium sp. MG2 as revealed by LC-MS and GC-MS.*

| Intermediate product | Results from LC-MS or GC-MS analysis | |
| --- | --- | --- |
|  | LC-MS | GC-MS |
|  | + | + |
|  | + | - |
|  | + | - |
|  | - | + |
|  | - | + |
|  | + | - |

+: detected. –: not detected.
